# Advanced oxidation protein products induce annulus fibrosus cell senescence through a NOX4-dependent, MAPK-mediated pathway and accelerate intervertebral disc degeneration

**DOI:** 10.7717/peerj.13826

**Published:** 2022-08-02

**Authors:** Xiangheng Dai, Yu Chen, Zihan Yu, Congrui Liao, Zhongyuan Liu, Jianting Chen, Qian Wu

**Affiliations:** Division of Spine Surgery, Department of Orthopaedics, Nanfang Hospital, Southern Medical University, Guangzhou, Guangdong, China

**Keywords:** Senescence, Intervertebral disc degeneration, Advanced oxidized protein products, Inflammation associated with senescence

## Abstract

**Background:**

Intervertebral disc degeneration (IVDD) is closely associated with senescence. Annulus fibrosus (AF) cell senescence is a crucial driver of AF tissue tearing and fissures, thereby exacerbating IVDD. Increased advanced oxidative protein products (AOPPs) were found in human degenerative discs and aged rat discs and may be involved in IVDD. This study aimed to explore the mechanism of AOPPs-induced senescence in AF cells.

**Methods:**

The pathological effects of AOPPs *in vivo* were investigated using a rat lumbar disc persistent degeneration model and a rat caudal disc puncture model. Rat primary AF cells were selected as *in vitro* models, and AOPPs were used as direct stimulation to observe their pathological effects. Setanaxb (NOX1/4 inhibitor), apocynin (NADPH oxidase inhibitor) and adenovirus (ADV) packed NADPH oxidase 4 (NOX4) specific shRNAs were used for pathway inhibition, respectively. Finally, adeno-associated viruses (AAVs) packed with NOX4-specific blocking sequences were used to inhibit the *in vivo* pathway.

**Results:**

AOPPs accumulated in the rat lumbar and caudal degenerative discs. Intra-discal loading of AOPPs up-regulated the expression of NOX4, p53, p21, p16, IL-1*β*, and TNF-*α*, ultimately accelerating IVDD. Exposure of AOPPs to AF primary cells up-regulated NOX4 expression, induced phosphorylation of mitogen-activated protein kinases (MAPK), triggered senescence and increased IL-1*β* and TNF-*α*. Apocynin, setanaxib, and ADV pre-cultured AF cells abrogated AOPPs-induced senescence. AAV-mediated inhibition of NOX4 expression *in vivo* reduced the expression of p53, p21, p16, IL-1*β* and TNF-*α in vivo* and delayed IVDD.

**Conclusions:**

AOPPs induced AF cell senescence through a NOX4-dependent and MAPK-mediated pathway.

## Introduction

Intervertebral disc degeneration (IVDD) is an ageing-related disease characterized by cell-mediated response to progressive structural failure and is regarded as a major cause of lower back pain (Luoma et al., 2000). IVDD is also a common and serious health problem, affecting more than 40% of those under 30 years old and more than 90% of those over 50 years old (Cheung et al., 2009). However, there are no effective therapies for IVDD because the pathogenesis of IVDD is still unclear. For patients with mild to moderate symptomatic IVDD who have failed conservative treatment and are not yet eligible for surgery, there is still a lack of effective treatment without side effects to relieve symptoms and preserve motor segments (Froud et al., 2014). Therefore, a better understanding of the pathogenesis of IVDD and its molecular pathways is essential to identify new therapeutic targets.

As an avascular organ, intervertebral discs consist of three interrelated structures: an inner central nucleus pulposus (NP), the outer surrounding annulus fibrosus (AF), and the cartilaginous and bony endplates. The main function of AF is mainly to bear the circumferential stress to suppress the tension generated by NP expansion and bending or twisting (Vo et al., 2016). AF cells play an important role in maintaining the stability of intervertebral disc. Age-related AF degeneration is characterized by loss of fibrous reticular network, increased number and size of fissures, and infiltration of blood vessels along with tears and cracks, with an increase in senescent AF cells (Patil et al., 2018). Cellular senescence is a typical feature of IVDD tissue and is thought to be involved in the development and progression of IVDD even in its early stage (Boos et al., 2002). However, there are few reports on AF cell senescence. Senescent disc cells can secretion of senescence-associated inflammatory proteins, such as IL-1*β* and TNF-*α*, which can not only regulate the secretion of matrix proteinase in non-aging disc cells, but also promote the aging of neighboring cells, and promote the infiltration of immune cells, leading to reduced matrix production and enhanced matrix protein degradation, which makes the disc tissue easy to be damaged (Patil et al., 2018). Eventually, IVDD is accelerated. Therefore, compounds that regulate cellular senescence might be perfect targets for IVDD therapy.

Oxidative stress is one of the main causes of cellular senescence (Brandl et al., 2011). Increased concentrations of oxidation products had been found in aged and degenerated IVDD tissues (Scharf et al., 2013; Sivan et al., 2006; Hou et al., 2014). Advanced oxidation protein products (AOPPs), a novel biomarker of oxidation-mediated protein damage (Wu et al., 2015), are involved in a variety of pathological changes, including our previous work on chondrocyte (Liao et al., 2020) and pro-osteoblast (Zhu et al., 2018) apoptosis, and levels of AOPPs have been reported to be much higher in degenerated (Pfirrmann IV or V) human IVDD tissue than in healthy samples (Xiang et al., 2020), which may suggest a pathogenic function for AOPPs in the progression of IVDD.

The pathological effects and downstream signaling pathways of AOPPs are mainly mediated by nicotinamide adenine dinucleotide phosphate (NADPH) oxidase-dependent reactive oxygen species (ROS) generation (Zhu et al., 2018; Cao et al., 2013). NADPH oxidase 4 (NOX4), in contrast to all other six members of the NADPH oxidase family, is constitutively active and produces relatively small amounts of ROS, maintaining the cellular redox state (Brandes, Weissmann & Schröder, 2014). NOX4-induced oxidative stress has been proven to be a major cause of NP cell senescence via the p53-p21-Rb and p16-Rb pathways (Feng et al., 2017). Moreover, AOPPs can up-regulate the expression of NOX4 in OA cartilage (Liao et al., 2020), activating downstream pathological effects. However, the role of the AOPPs/NOX4 pathway in AF cell senescence is unclear.

This study aimed to test the hypothesis that the AOPPs/NOX4 pathway may induce senescence and inflammatory responses in AF cells. We demonstrated that AOPPs up-regulated the expression of senescence marker p16 and p21 in AF cells, and induced the expression of senescence-associated inflammatory protein IL-1*β* and TNF-*α*, mediated mainly by a NOX4-MAPK dependent redox pathway. These results reveal novel mechanisms of AF cell senescence activation and provide a link between oxidative protein damage and IVDD inflammatory progression.

## Material and Methods

### Ethics approval

All the animal experimental procedures had been approved by the Laboratory Animal Care and Use Committee of Nanfang Hospital, Southern Medical University (NFYY-2020-78). All the steps described in this study meet the standards of the Eighth Edition Guidelines for the Care and Use of Laboratory Animals published by the National Academy of Sciences (Washington, DC, USA).

### Preparation of AOPPs-RSA

AOPPs-RSA were prepared based on the procedure described previously (Zhu et al., 2018). In brief, RSA (Rat Serum Albumin; HUIJIA Biotechnology, China) solution (20 mg/ml) was incubated with 40mM HOCl in phosphate-buffered saline (PBS, pH = 7.4) for 30 min at room temperature. Prepared samples were dialyzed for 24 h against PBS that change every 6 h to remove free HOCl. Native RSA was dissolved in PBS alone as control incubation.

### Animal and experimental design

Thirty-six adult male Sprague–Dawley (SD) rats(500 ± 50 g) were randomized into six groups. Caudal discs were exposed without puncture for the sham-operated control (Group 1). Under anesthesia, two segmental caudal discs (Co6/7, Co8/9) were exposed through a posterior incision. Disc puncture was performed using a 21-gauge needle each on Co6/7, Co8/9 to cause IVDD, as mentioned previously (Group 2) (Keorochana et al., 2010; Zhang et al., 2011). To make sure that the needle did not penetrate too deeply, the length of the needle was pre-determined by approximately five mm, as reported previously (Keorochana et al., 2010; Zhang et al., 2011). Untreated Co7/8 served as the internal control. Based on our previous work demonstrates that intra-articular injections of AOPPs in rabbit OA models (Yu et al., 2015) and intraperitoneal injection in mice OA models (Liao et al., 2020) accelerate regression of cartilage, we designed Group 3 was given intra-peritoneal AOPPs (100 mg/kg/d) for 1 month and Group 4 was given intra-peritoneal AOPPs (100mg/kg/d) 3 days after disc puncture for 1 month. To evaluate the effect of AOPPs on AF cell senescence and IVDD *in vivo*, we prepared two solutions for intra-discal injection, including RSA (50 µg/ml) (Group 5) and AOPPs (200 µg/ml)(Group 6). 2 µl of the solution of interest was injected into each segment, and 30s were kept in the disc for each 31-gauge needle (Mao et al., 2011). The injections were conducted once every fortnight for 1 month, respectively. After surgery, each animal was allowed free, unrestricted weight-bearing and activity, fed normally, and monitored. X-ray (FX Pro, Bruker, SMU Central Laboratory, Southern Medical University) was performed 1 month after surgery for each group to measure disc height. Caudal discs were harvested for western blotting and immunohistochemical staining.

Eighteen adult male SD rats (500 ± 50 g) were randomized into three groups. In the Sham group, only the skin on the back of the lumbar vertebrae of the rats was cut and sutured. In the degeneration group, the lamina, zygapophyseal joint, and spinous process of the 1-6 lumbar vertebrae were excised, and MRI (PharmaScan70/16 US, Bruker, SMU Central Laboratory, Southern Medical University) was performed at 1 and 2 months postoperatively to examine the degeneration of the lumbar spine, and the lumbar disc was finally removed for western blotting and immunofluorescence.

Twenty-four adult male SD rats (500 ± 50 g) were randomized into four groups. *In vivo* blockade of NOX4 expression was achieved by using Adeno-Associated Viruses (AAV) that were purchased from the Shanghai OBiO Technology carrying the rat NOX4 shRNA-2 sequences that can block NOX4 expression and injecting 2 µl AAV into the intervertebral disc using a 31 g needle. AAV carrying the NC sequence was used as a control. RSA and AOPPs were re-injected into the intervertebral disc 3 weeks after injection, once every fortnight for 1 month, respectively. MRI was performed 1 month after surgery for each group to measure Pfirrmann grade. Caudal discs were harvested for western blotting and immunofluorescence.

### Immunohistochemical staining and immunofluorescence

After 24–48 h of paraformaldehyde fixation, discs tissues were decalcified for at least eight weeks with Ethylene Diamine Tetraacetic Acid (EDTA). Then after dehydrated and paraffin-embedded, samples were sectioned to 4 µm. After being treated with 0.3% hydrogen peroxide for 10 min to reduce the activity of endogenous peroxidase, nonspecific staining was blocked by incubating the sections with 10% goat serum for 30 min. Then slices were incubated with primary antibodies against NOX4 (1:50, Affinity, DF6924), p16 (1:100, Abclonal, A0262), AOPPs (1:100, Department of Immunology, Southern Medical University), p21 (1:100, CST, #2946), p53 (1:100, Abclonal, A11232), IL-1*β* (1:100, Affinity, AF5103), TNF-*α* (1:100, Affinity, AF7014), p-p38 (1:100, Affinity, AF4001), p-ERK (1:100, Affinity, AF1015), p-JNK (1:100 dilution, Affinity, AF3318) at 4 °C overnight, followed by incubated with HRP-conjugated secondary antibodies or Cy3 fluorescent secondary antibody for 1 h at room temperature. DAB Horseradish Peroxidase Color Development Kit(Beyotime) was used for immunohistochemical positive cells color rendering. Nuclei were counterstained with hematoxylin or DAPI. All sections were viewed examined with a Leica DM5000B (Leica, Germany) and ZEISS AXIO Imager D2 (Germany). Fluorescent images are taken at the same exposure time for each group of target proteins, and the grey scale values are automatically measured by Imager D2.

### Rat AF cell isolation and culture

As reported previously (Zhao & Tian, 2019), AF tissue was separated under a dissecting microscope and then was digested using 0.1% Type II collagenase (Sigma-Aldrich, St. Louis, MO, USA) for 5 h with shaking at 37 °C. The isolated AF cell pellets were re-suspended in DMEM/F12 medium (Gibco, Waltham, MA, USA) containing 10% fetal bovine serum (FBS, Gibco, Waltham, MA, USA) and 1% (v/v) penicillin-streptomycin (Gibco, Waltham, MA, USA), then seeded onto a 21 cm^2^ culture dish at 37 °C in a humidified atmosphere of 5%CO_2_–95%air. Up to 80%–90% confluence, the second or third passage AF cells were used in each assay.

### Western blot analysis

Total proteins of AF cells or disc AF tissues were extracted using a protein extraction reagent (FUDE Biological Technology) and quantified using a BCA kit(Thermo Scientific). Proteins mixed with 1/4 volume loading buffer (FUDE Biological Technology) were electrophoresed on SDS gels and then were transferred to PVDF membranes (Millipore). The membranes were blocked by using 5% Bovine Serum Albumin in TBST for 1 h at room temperature. Next, the membranes were incubated with primary antibodies against GAPDH (1:1000, Bioworld, BS60630), p16 (1:500, Abclonal, A0262), p21 (1:1000, CST, #2946), AOPPs (1:1000, Department of Immunology, Southern Medical University), Albumin (1:500, Affinity, DF6396), p53 (1:500, Abclonal, A11232), p38 (1:500, Affinity, AF6456), p-p38 (1:500, Affinity, AF4001), ERK1/2 (1:500, Affinity, AF0155), p-ERK1/2 (1:500, Affinity, AF1015), JNK (1:500, Affinity, AF6318), p-JNK (1:500, Affinity, AF3318), IL-1*β* (1:500, Affinity, AF5103), TNF-*α* (1:500, Affinity, AF7014) and NOX4 (1:500, Affinity, DF6924) with slowly shaking overnight at 4 °C, followed by incubation with the HRP-conjugated secondary antibodies (1:5000 dilution, Abmart, M21003) at room temperature for 1 h. Immunolabeling was detected using an ECL kit (Affinity, KF001). The total optical density (OD) of blot bands was measured by using Image J software (National Institutes of Health, Bethesda, MD, USA). The relative expression of each protein = OD of each protein / OD of each GAPDH. The relative phosphorylation level of each protein = OD of each phosphor-protein/ OD of each total protein.

### SA-*β*-gal staining

The SA-*β*-gal staining of AF cells was investigated using the SA-*β*-gal staining kit (Solarbio). According to instruction, fixative AF cells seeded in 6-well culture plates were incubated with the staining solution at 37 °C overnight. Nine random fields per well were imaged using a phase-contrast microscope (Olympus) to calculate the mean percentage of SA-*β*-gal positive cells.

### Cell cycle analysis

To determine cell cycle distribution, NP cells were re-suspended in ice-cold 70% ethanol and incubated at 4 °C overnight. The next day, the cells were washed with PBS again and stained with propidium iodide (PI) using the cell cycle and apoptosis analysis kit (Beyotime, C1052) for 30 min. Cells were analyzed using flow cytometry (BD FACSCalubur™, USA) and cell proportions (in%) were measured using FlowJo7.6.1 software. Gate strategy: gate 1, horizontal coordinate (FSC), vertical coordinate (SSC), circles out the main population of cells based on their forward and lateral angles. Then put this group of cells in Gate 2, horizontal coordinate (PI-W), vertical coordinate (PI-A), and circle out the target cells according to the width and peak of PI. Finally, put this cluster of cells in (PI-A) and divide it into three phases according to the amount of PI staining.

### Transfections of adenovirus

Adenovirus with shNOX4 and adenovirus with NC sequences were purchased from the Shanghai OBiO Technology. The sequence of rat NOX4 shRNA-1 is GCTTCTACCTATGCAATAA. The sequence of rat NOX4 shRNA-2 is GCAACAAACCTGTCACCAT. The sequence of rat NC shRNA is CCTAAGGTTAAGTCGCCCTCG. Transfections of adenovirus into AF cells were according to the manufacturer’s instructions. Approximately 72 h after transfection, virus delivery was determined by observing green fluorescent protein (GFP) under the fluorescence microscope (Olympus). The ratio of GFP-positive cells to total cells was used to define transfection efficiency. Cells with a transfection efficiency of more than 90% were used in each assay. Cell lysates were collected, and Western blotting was used to examine their efficiency.

### Dimethylmethylene blue

For GAGs level determination, total protein extract from disc tissue was measured using a 1,9-dimethyl methylene blue (DMMB) method with Bovine chondroitin 4-sulfate(Sigma-Aldrich) as a standard. 200 µl DMMB solution was added to the wells on a 96-well plate containing 40 µl of a sample. The DMMB solution was made by mixing 16 mg DMB, 3.04 g glycine, 1.6 g NaCl and 95 ml of 0.1 M Acetic Acid in 1 L of distilled water. The absorbance was measured at *λ* = 525nm on a SpectraMax M5 system (Molecular Devices, San Jose, CA, USA).

### Statistical analysis

All experiments were repeated independently at least three times and the data are presented as the mean ± SD. All statistical analyses were performed by using Statistical Packages for Social Sciences v20.0 software (SPSS, Chicago, IL). Results for continuous variables such as grey values of western blot and immunofluorescence, SA-*β*-gal Staining, Cell cycle analysis, and Dimethyl methylene blue were analyzed using one-way ANOVA. Nonparametric data like disc Pfirrmann grade were analyzed with the Kruskal-Wallis H test. Post hoc two-by-two comparison using adjusted *α* value. *P* ≤ 0.05 was considered significant.

## Results

### AOPPs accumulate in IVDD, up-regulate the expression of NOX4, senescence markers, and senescence-associated inflammatory proteins, and accelerate IVDD

To verify whether AOPPs are involved in the course of natural IVDD, we constructed a model of persistent lumbar disc degeneration in rats. A model of lumbar instability was constructed by removing the posterior bony structures of the lumbar 1-6 vertebrae, *i.e.*, the lamina, zygapophyseal joint, and spinous process, while preserving muscle tissue to avoid excessive damage to the lumbar mobility of the rat, thereby accelerating IVDD without directly damaging the disc.

**Figure 1:**
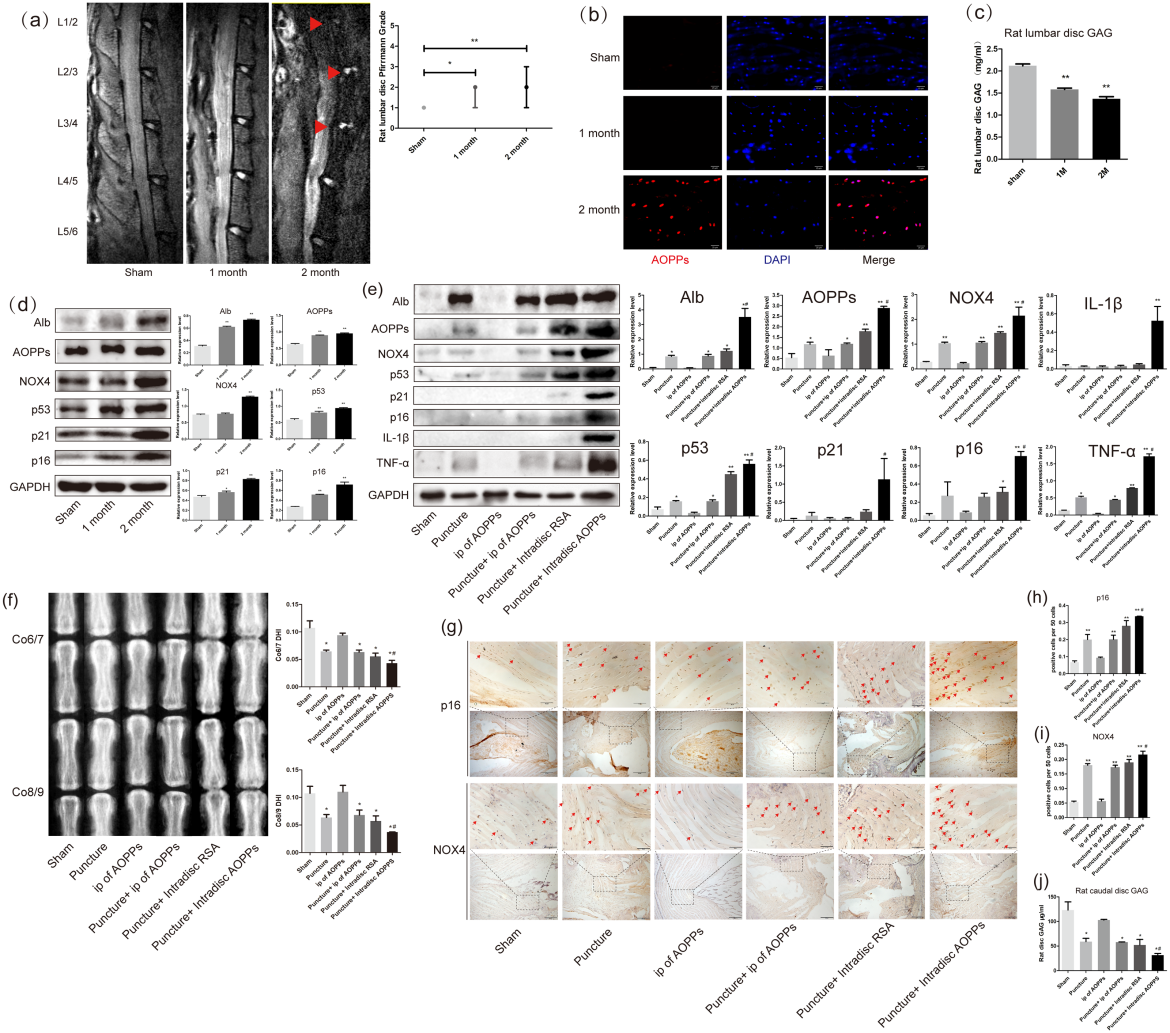
AOPPs accumulate in IVDD, up-regulate the expression of NOX4, senescence markers, and their associated inflammatory proteins, and accelerate IVDD. (A) MRI of a persistent lumbar degeneration model showed significant disc degeneration. (B) Immunofluorescence of AOPPs showed massive accumulation of AOPPs in AF tissues at two months of degenerative discs. Scale bar: 25 µm. (C) The DMMB assay of the rat lumbar discs showed a gradual decrease in GAG content after surgery, suggesting IVDD. (D) Western blot analysis of lumbar discs showed that albumin and AOPPs accumulated in IVDD, up-regulating NOX4, senescence markers p53, p21, p16, and senescence-associated inflammatory proteins IL-1*β* and TNF-*α* expression. (E) Western blot analysis of the caudal discs showed that albumin and AOPPs accumulated in IVDD up-regulated the expression of NOX4, senescence markers, and senescence-associated inflammatory proteins. (F) X-rays of Co6/7 and Co8/9 showed lower DHI in the puncture + intra-discal AOPPs group. (G, H, I) Immunohistochemical staining of AF tissue for p16 and NOX4 was positive in the punctured discs and much higher after stimulation with exogenous AOPPs. Scale bars: 100 µm and 200 µm. (J) DMMB measurements of rat lumbar discs showed a decrease in GAG content in all puncture groups, indicating IVDD in all puncture groups, with the lowest content and most severe degeneration in the intra-discal injection AOPPs group. Sham, sham surgery. IP, intra-peritoneal injection. *N* = 6 per group. *P < 0.05 compared to Sham and IP for AOPPs. ** *P* < 0.01 compared to Sham and IP for AOPPs. #*P* < 0.05 compared to the other five groups. All experiments were repeated at least three times. Error bars represent standard errors.

**Figure 2:**
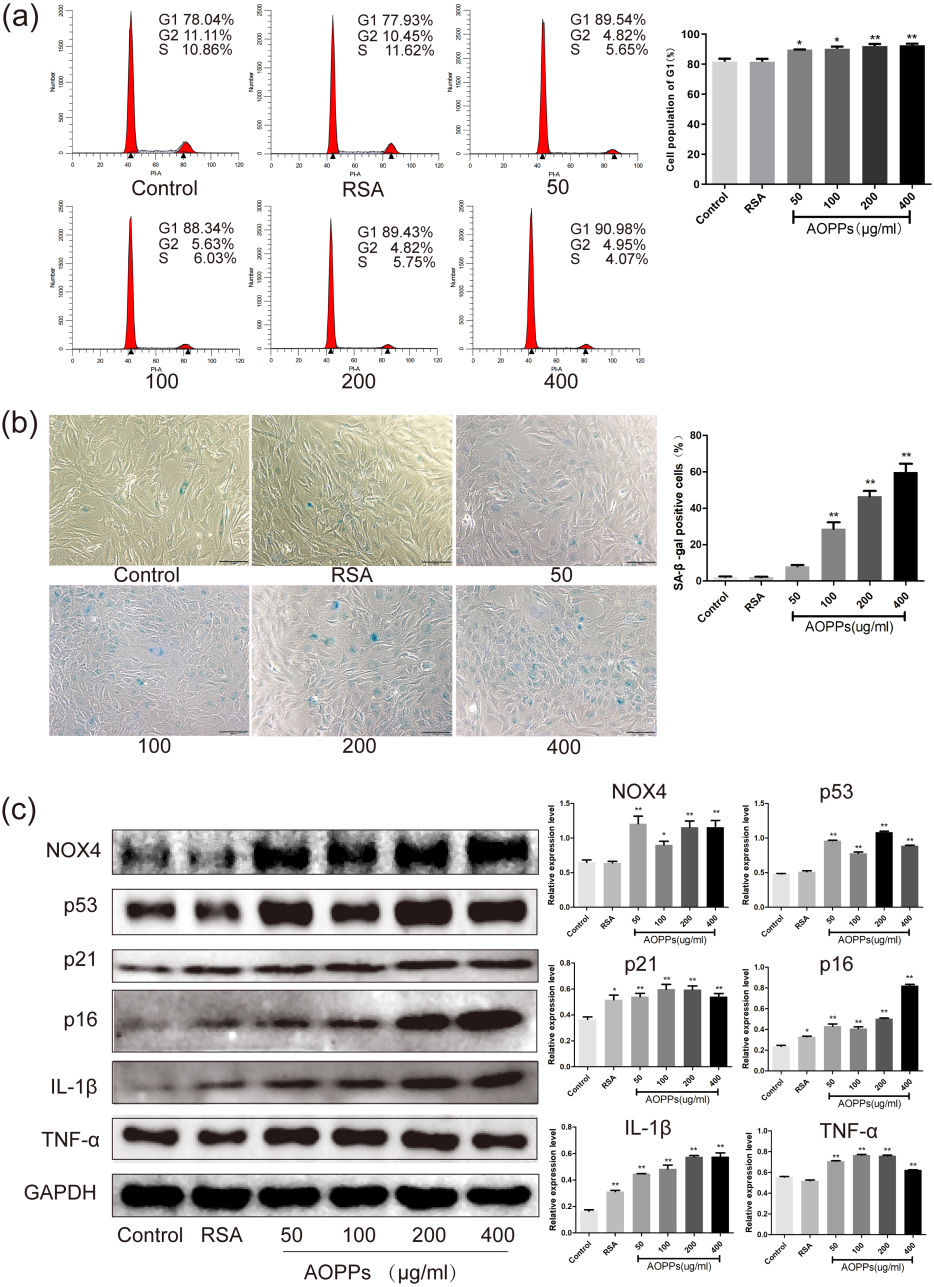
AOPPs up-regulate NOX4 expression, induce senescence in AF cells, and increase the secretion of senescence-associated inflammatory proteins. (A) FACS analysis of AF cells treated with AOPPs (50–400 µg/mL, 24 h) induced G1 phase arrest. (B) SA-*β*-gal staining of AF cells treated with AOPPs (50–400 µg/mL, 24 h). AOPPs increased the proportion of senescent AF cells. Scale bar: 100 µm. (C) AOPPs significantly increased the expression of NOX4, p53, p21, p16, IL-1*β* and TNF-*α* in AF cells. *P < 0.05 compared to the control. **P < 0.01 compared to the control. Error bars represent standard errors.

As shown in Fig. 1A, after resection of the posterior lumbar structures, we found significant degeneration of the lumbar 1/2,2/3 and 3/4 discs, while the lumbar 4/5 and 5/6 discs showed protrusion into the spinal canal at two months after surgery. In Fig. 2C, the results of the DMMB suggested that the GAG content of the lumbar intervertebral discs decreased significantly from one month postoperatively and dropped to a minimum in two months postoperatively, suggesting the lumbar IVDD in rats. Immunofluorescence experiments in Fig. 2B showed a significant accumulation of AOPPs in the degenerated AF tissues at two months after surgery. In subsequent WB analysis, we found that albumin, which could be oxidized to AOPPs, also accumulated in the degenerated lumbar discs. At the same time, AOPPs, NOX4, senescence markers: p53, p21, and p16, as well as senescence-associated inflammatory proteins: IL-1*β* and TNF-*α* were highly expressed in the degenerated discs (Fig. 1D).

From the mechanism of AOPPs formation and the above experimental results, we can speculate that AOPPs may not be the initiating factor in disc degeneration and that their pathological effects may be more focused on accelerating disc degeneration. To test this conjecture, we constructed a rat caudal disc puncture model. Interestingly, after being punctured, the albumin was accumulated in the degenerative discs. As a result, AOPPs were also accumulated, accompanied with high expression of NOX4, p53, p21, p16, IL-1*β*, and TNF-*α*, especially higher in the intra-discal injection of AOPPs group (Fig. 1E). In Fig. 1F, the radiographs showed that the disc height index (DHI) of the punctured discs were all low, suggesting disc space collapse and significant disc degeneration, with the lowest DHI in the intra-discal injection AOPPs group. The dimethylmethylene blue assay (DMMB) of the disc tissue showed (Fig. 1J) that the GAG were lower in all groups of discs after the puncture, with the lowest levels in the intra-discal injection AOPPs group. Based on the results of DHI and DMMB, it’s obvious that degeneration was observed in all puncture groups, and the intra-discal AOPPs injection caused the most serious damage, while the intra-peritoneal(ip) AOPPs injection could not cause more damage no matter puncture or not. Finally, in the AF tissue immunohistochemistry staining, we detected the expression of p16 (Figs. 1G and 1H) and NOX4 (Figs. 1G and 1I) that was positive in puncture discs and much higher after exogenous AOPPs stimulation.

### AOPPs up-regulate NOX4 expression, induce senescence in AF cells, and increase the secretion of senescence-associated inflammatory proteins

**Figure 3:**
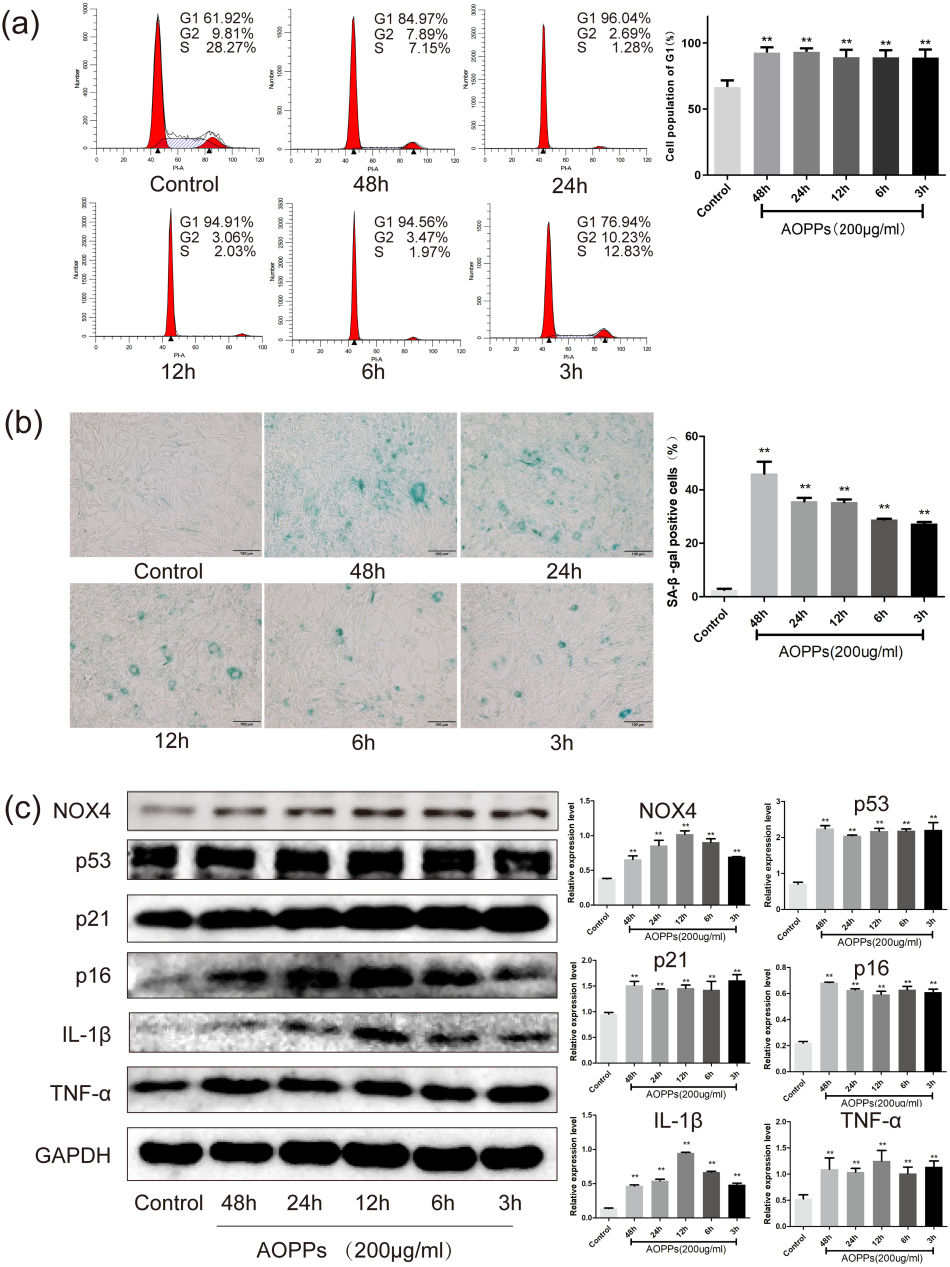
AOPPs up-regulate NOX4 expression, induce senescence in AF cells, and increase the secretion of senescence-associated inflammatory proteins. (A) FACS analysis of AF cells treated with AOPPs (200 µg/mL, 3–48 h) to induce G1 phase arrest. (B) SA-*β*-gal staining of AF cells treated with AOPPs (200 µg/mL, 3–48 h). AOPPs increased the proportion of senescent AF cells. Scale bar: 100 µm. (C) AOPPs significantly increased the expression of NOX4, p53, p21, p16, IL-1*β* and TNF-*α* in AF cells. *P < 0.05 compared to the control. **P < 0.01 compared to the control. Error bars represent standard errors.

Based on the above findings, we used rat AF primary cells as an *in vitro* model to investigate whether the accumulation of AOPPs directly triggers the senescence of AF cells. We first studied the cell cycle distribution of AF cells by quantitative fluorescence-activated cell sorting (FACS) analysis. AOPPs were used to treat cells at varying doses (50–400 µg/mL, 24 h) and time (200 µg/mL, 3-48 h), and it was found that the proportion of AF cells arrested in G1 phase arrest was increased (Figs. 2A and 3A). We further used SA- *β*-gal staining to confirm senescence. As shown in Figs. 2B and 3B, AOPPs increased the proportion of senescent AF cells at different concentrations and times. Similarly, expression of NOX4, p53, p21, p16, IL-1*β*, and TNF-*α* was up-regulated by AOPPs treatment at different doses and times (Figs. 2C and 3C).

### The AOPPs-induced senescence of AF cells is mediated by NADPH oxidase

**Figure 4:**
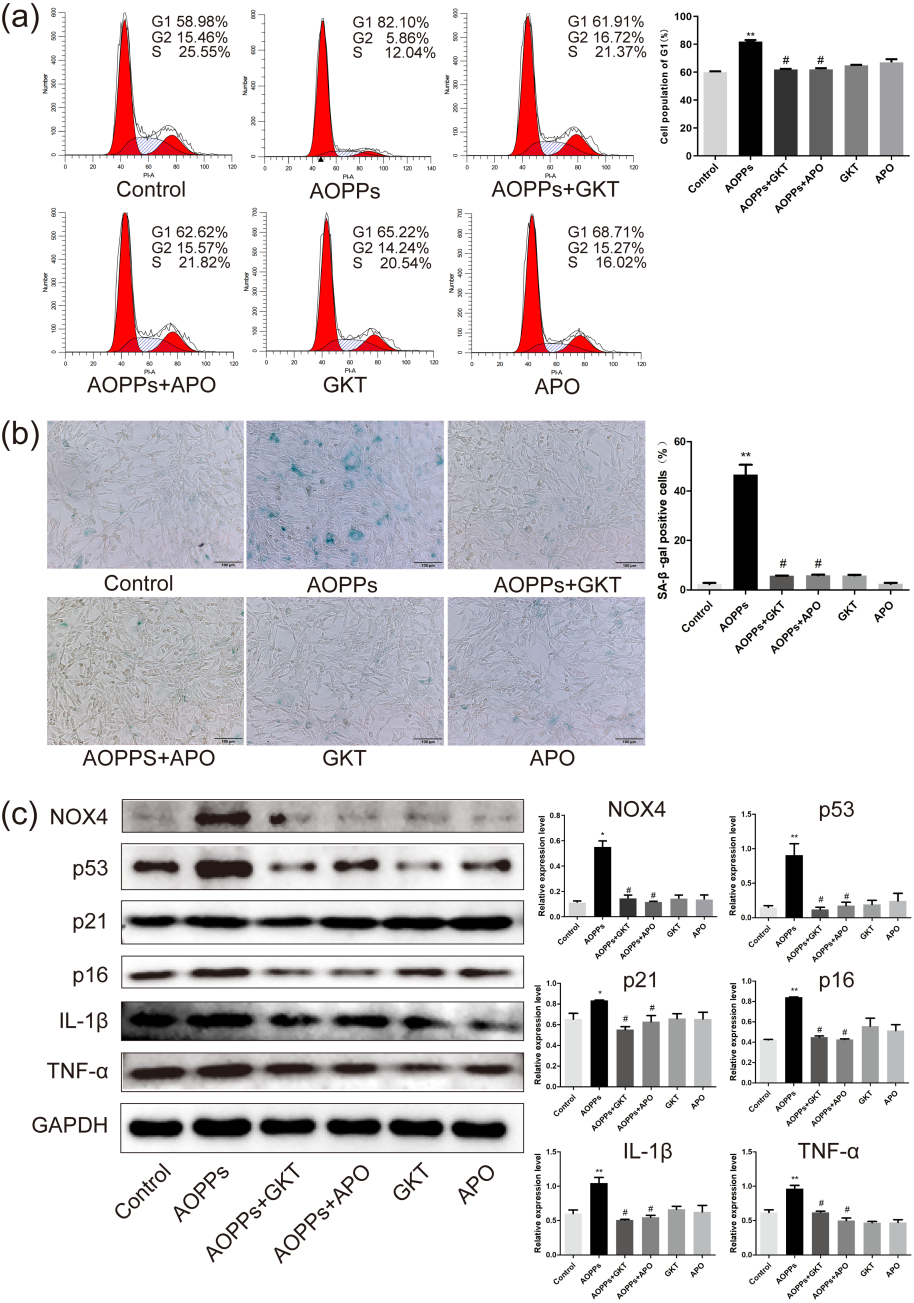
The AOPPs-induced senescence of AF cells was mediated by NADPH oxidase. (A) FACS analysis of AF cells pre-incubated with GKT (20 µM) and APO (100 µM) prevented AOPPs-induced G1 phase arrest. (B) SA-*β*-gal staining of AF cells pre-incubated with GKT (20 µM) and APO (100 µM) showed restoration of AOPPs-induced senescence. Scale bar: 100 µm. (C) Expression of NOX4, p53, p21, p16, IL-1*β* and TNF- *α* was down-regulated in response to inhibitor treatment. *P < 0.05 compared to the control. **P < 0.01 compared to the control. #*P* < 0.05 compared to the AOPPs group. Error bars represent standard errors.

Previous studies in our group suggest that NADPH oxidase was the main effector molecule of AOPPs (Liao et al., 2020; Zhu et al., 2018). To confirm the role of NADPH oxidase in AOPPs-induced AF cell senescence, we pre-incubated cells with two NADPH oxidase inhibitors: Setanaxib (GKT137831, Selleck) (GKT) and Apocynin (NSC2146, Selleck) (APO), before AOPPs exposure, respectively. Blocking down NADPH oxidase restored the AOPPs-induced G1 phase arrest (Fig. 4A), as well as the positive cells of SA-*β*-gal staining (Fig. 4B). Similarly, High expression of NOX4, p53, p21, p16, IL-1*β* and TNF-*α* induced by AOPPs was down-regulated in response to inhibitor treatment (Fig. 4C).

### AOPPs induce AF cell senescence by activating phosphorylation of MAPK

**Figure 5:**
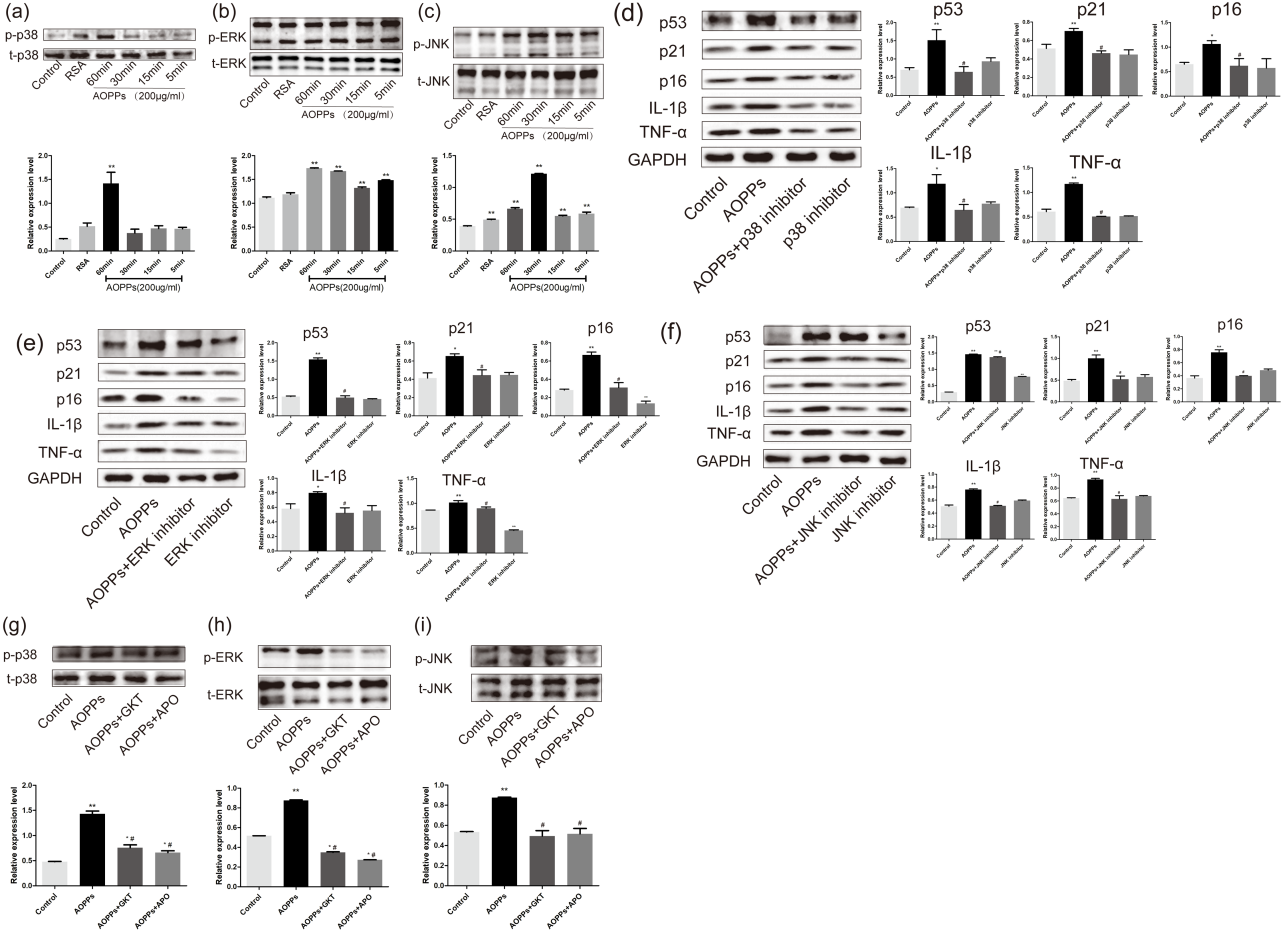
AOPPs induce AF cell senescence by activating phosphorylation of MAPK. (A–C) AOPPs (200 µg/mL, 24 h) significantly increased phosphorylation of p38, ERK1/2 and JNK in AF cells. (D–F) p38 MAPK inhibitor (SB202190, 10 µM), ERK1/2 inhibitor (FR180204, 10 µM) and JNK inhibitor (SP600125, 10 µM) AOPPs-induced (200 µg/mL, 24 h) senescence in AF cells. (G–I) Activation of AOPPs-induced (200 µg/mL, 24 h) MAPK phosphorylation was ameliorated by GKT (20 µM) and APO (100 µM). *P < 0.05 compared to the control. ** *P* < 0.01 compared to the control. #*P* < 0.05 compared to the AOPPs group. Error bars represent standard errors.

Previous studies in our group suggested MAPK were major effector molecules in the pathology mediated by AOPPs (Liao et al., 2020; Zhu et al., 2018). To determine whether the MAPK pathway is involved in AOPPs-induced senescence, we evaluated MAPK phosphorylation in AF cell cultures treated with AOPPs. Our findings revealed that phosphorylation of p38 MAPK appeared after 60 min of AOPPs stimulation, while ERK phosphorylation appeared 5 min after AOPPs stimulation, and JNK phosphorylation similarly began to rise 5 min after AOPPs stimulation, while AF cells stimulated with RSA for 60 min showed a slight rise in JNK phosphorylation (Figs. 5A, 5B and 5C). To further evaluate the role of the MAPK pathway in senescence, AF cell cultures were incubated with a p38 MAPK inhibitor (SB202190; Beyotime, Shanghai, China), an ERK1/2 inhibitor (FR180204; Beyotime, Shanghai, China), a JNK inhibitor (SP600125; Beyotime, Shanghai, China) respectively, before AOPPs treatment. Our findings revealed that the AOPPs-induced senescence in AF cells was ameliorated by all three inhibitors (Figs. 5D, 5E and 5F).

To better confirm that NADPH oxidase was activated earlier than MAPK, AF cells were incubated with GKT and APO before AOPPs treatment. Our findings showed activation of AOPPs-induced MAPK phosphorylation was ameliorated by GKT and APO (Figs. 5G, 5H and 5I).

### AOPPs induce AF cell senescence via the NOX4-MAPK pathway

**Figure 6:**
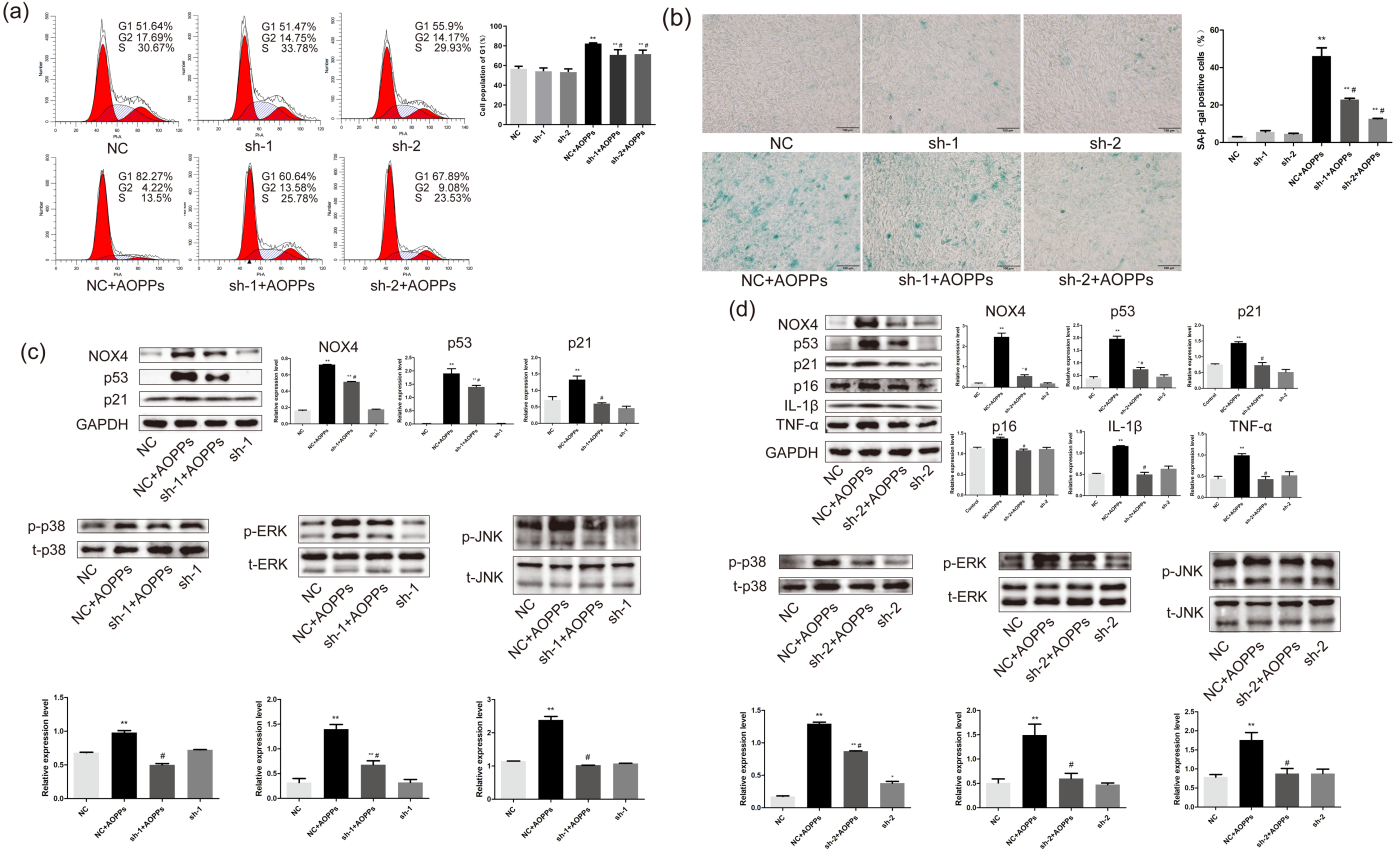
AOPPs induce AF cell senescence via the NOX4-MAPK pathway. (A) NOX4-specific shRNAs significantly inhibited AOPPs-induced (200 µg/mL, 24 h) G1 phase arrest. (B) NOX4-specific shRNAs significantly reduced AOPPs-induced (200 µg/mL, 24 h) SA-*β*-gal staining of positive cells. Scale bar: 100 µm. (C) NOX4 shRNA-1 only blocked the expression of p53, p21, and the hyperphosphorylation levels of p38, ERK, and JNK, but not the expression of p16, IL-1*β*, and TNF-*α*. (D) AOPPs-induced (200 µg/mL, 24 h) up-regulation of NOX4, p53, p21, p16, IL-1*β*, and TNF-*α*, and hyperphosphorylation levels of p38, ERK, and JNK were reduced after transfection with NOX4-specific blockade of shRNA-2 sequences. *P < 0.05 compared to NC. **P < 0.01 compared to NC. #*P* < 0.05 compared to NC+AOPPs. Error bars represent standard errors.

Based on the findings that both GKT and APO can ameliorate the AOPPs-induced senescence, and as NOX1 is mainly presented in colon epithelium (Szanto et al., 2005), we decided to further confirm the role of NOX4 in AOPPs-induced senescence in AF cells. We pre-incubated cells with two NOX4-specific shRNAs packaged by adenoviruses before AOPPs exposure. NC shRNA packaged by adenoviruses was used as a control. As shown in Fig. 6A, NOX4-specific shRNAs significantly inhibited AOPPs-induced G1 phase arrest. The positive cells of SA-*β*-gal staining were reduced (Fig. 6B). AF cells transfected with ADV packed with NOX4-specific blocking shRNA sequences did not show cell cycle arrest and increased positive staining for senescence, suggesting that the transfected AF cells were in good condition. As shown in Fig. 6C, NOX4 shRNA-1 only blocked p53 and p21, but not p16, IL-1*β* and TNF-*α* expression (data not shown), whereas as shown in Fig. 6D, NOX4 shRNA-2 was well blocked by both. AOPPs-induced up-regulation of NOX4, p53, p21, p16, IL-1*β*, TNF-*α*, and hyper-phosphorylation levels of p38, ERK, and JNK were reduced after transfection with NOX4-specific blockade of shRNA-2 sequences (Figs. 6C and 6D).

### Blocking NOX4 expression *in vivo* by AAV reduces the expression of senescence markers and senescence-related proteins and delays IVDD

**Figure 7:**
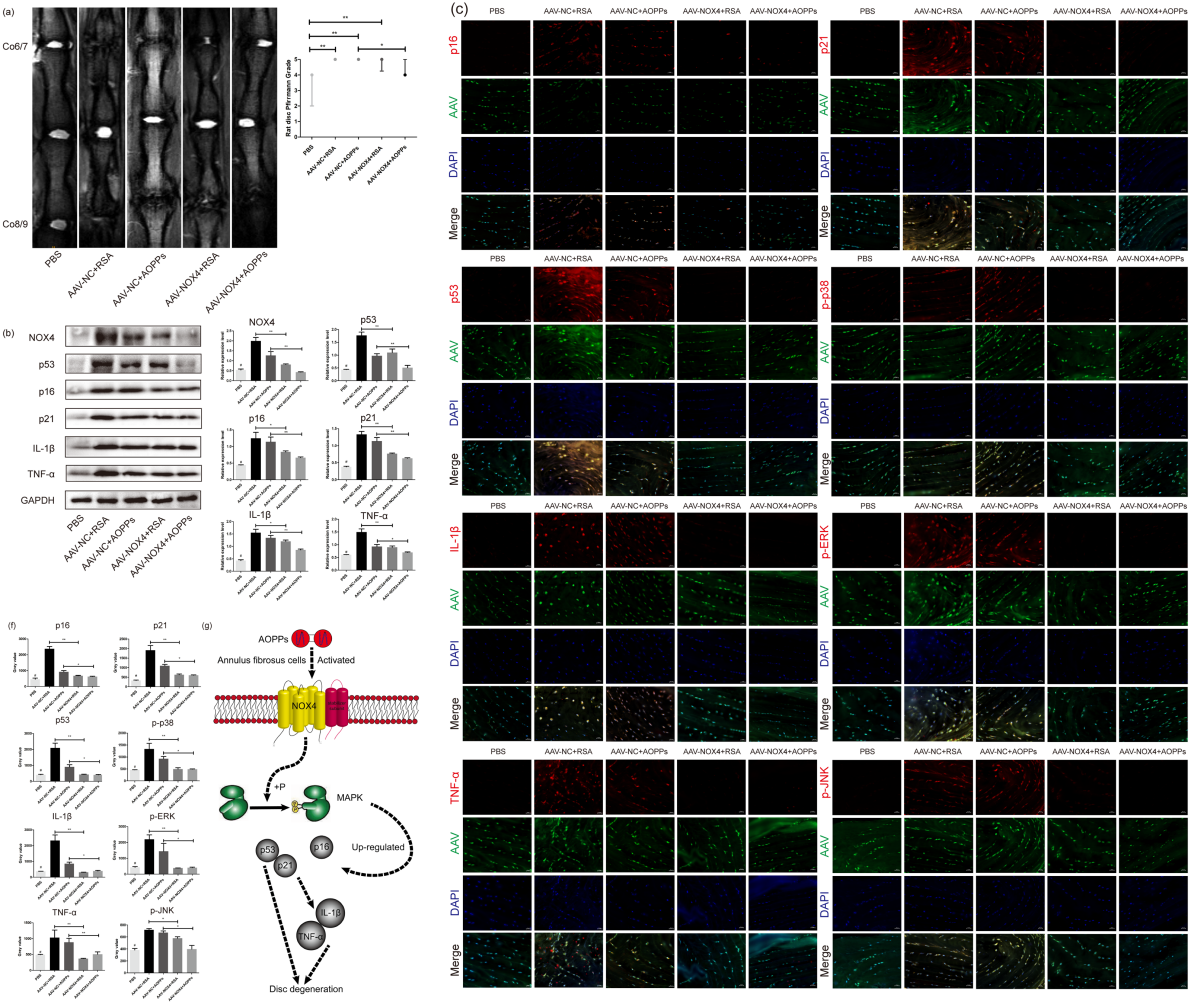
Blocking NOX4 expression *in vivo* by AAV delays IVDD. (A) MRI showed that blocking NOX4 expression by AAV reduced the Pfirrmann grade of disc degeneration caused by RSA and AOPPs. *P < 0.05. **P < 0.01. Error bars represent the interquartile range. (B, C) HE staining and saffron O/fast green staining showed less disc destruction caused by AOPPs and RSA in discs with blocked NOX4 expression and correspondingly decreased disc injury scores. **P < 0.01 and #*P* < 0.05 compared with the other four groups. (D) Western blot analysis showed that blocking NOX4 expression reduced the expression of p53, p21, p16, IL-1*β* and TNF-*α* in disc tissue injected with AAV. (E, F) Immunofluorescence assay showed that blocking NOX4 expression reduced the expression of p53, p21, p16, p-p38, p-ERK, p-JNK, IL-1*β* and TNF-*α*. (G) Diagram of AOPPs-induced NOX4, p53, p21, p16, IL-1*β* and TNF-*α* expression. #*P* < 0.05 compared to the AAV-NC+RSA and AAV-NC+AOPP groups. *P < 0.05. **P < 0.01. Error bars represent standard errors.

As suggested by the MRI in Fig. 7A, *in vivo* blocking NOX4 expression by AAV reduced the Pfirrmann grade of disc degeneration caused by RSA and AOPPs and delayed IVDD. HE staining and staining with saffron O/fast green showed that in discs with blocked NOX4 expression, disc damage caused by injections of AOPPs and RSA was less severe, the growth plate and part of the cartilage endplate were preserved, the morphology of the AF tissue was improved, the number of surviving AF cells was higher and, overall, the corresponding disc damage scores were lower (Figs. 7B and 7C). Furthermore, blocking NOX4 expression before stimulation with RSA and AOPPs reduced the expression of p53, p21, p16, p-p38, p-ERK, p-JNK, IL-1*β*, and TNF-*α* in the disc (Figs. 7D, 7E and 7F).

## Discussion

AF tissue is an indispensable supporting component to protect NP tissue and to maintain the regular biomechanical function of the disc. Previous studies have reported that tears and fissures of AF tissue are closely related to the occurrence and aggravation of IVDD (Nerurkar et al., 2009), which the potential mechanism is AF cell senescence (Zhao & Tian, 2019). In the current study, we provided both *in vitro* and *in vivo* evidences that the senescence of AF cells may be triggered by AOPPs/NOX4 pathway. *In vivo*, we demonstrated that AOPPs were accumulated in the rat lumbar and caudal degenerative discs, accompanying up-regulated expression of NOX4, senescence markers, and senescence-associated inflammatory proteins. *In vitro*, we further figured out that AOPPs enhanced the expression of p53, p21, p16, IL-1*β*, and TNF-*α* in rat AF primary cells via the NOX4-dependent, MAPK mediated pathway.

AOPPs are a family of di-glycine-containing protein products formed by the reaction of plasma proteins such as albumin with hypochlorous acid (Cao et al., 2013). To verify whether AOPPs are involved in the pathogenesis of natural disc degeneration, we constructed a model of persistent lumbar degeneration in rats. A model of lumbar instability was constructed by removing the posterior structures of the rat lumbar spine 1-6, *i.e.*, the lamina, zygapophyseal joint, and spinous process, while preserving muscle tissue to avoid excessive damage to the lumbar mobility of the rat, thereby accelerating disc degeneration without directly damaging the disc. We found that AOPPs were present in the degenerated discs and showed significant accumulation at 2 months postoperatively. Similarly, albumin also showed accumulation in the degenerated discs, further increasing the likelihood that AOPPs were present in the degenerated discs. NOX4 and senescence-related molecules were also highly expressed 2 months after surgery, suggesting that AOPPs may be involved in the natural course of IVDD by inducing senescence.

From the mechanism of AOPPs formation and the above experimental results, we can deduce that AOPPs may not be the initiating factor of disc degeneration, as AOPPs need to be converted from albumin in an environment of oxidative stress, so their pathological effects may be more focused on accelerating disc degeneration. To verify this, we further constructed a rat caudal disc puncture model and subsequently loaded it with RSA and AOPPs, while setting up an intra-peritoneal AOPPs injection group to understand whether elevated peripheral blood AOPPs levels affect disc degeneration. First, we found an accumulation of albumin and AOPPs in the disc degeneration caused by the puncture, further suggesting the involvement of AOPPs in the pathological process of disc degeneration. Subsequently, NOX4, senescence markers, and inflammation-related factors were also highly expressed, being highest in the group loaded with AOPPs after the puncture; the results of GAGS content measured by DMMB and DHI measured by X-ray were lowest in the group loaded with AOPPs after the puncture, suggesting that an increase in AOPPs in the disc accelerates disc degeneration. In contrast, the intra-peritoneal injection of AOPPs group, whether punctured or not, did not significantly elevate the content of AOPPs within the disc, and none of the damage to the disc was significant, suggesting that the increase in the content of peripheral AOPPs did not accelerate disc degeneration. This may be because most of the AOPPs injected intra-peritoneally can be rapidly metabolized by metabolic organs such as the liver, spleen, and kidney (Iwao et al., 2006).

Our previous study demonstrated that AOPPs was increased in aged rat disc (Hou et al., 2014), which may participate in age-related disc changes. Herein, AOPPs-induced senescence was then confirmed by the *in vivo* study, in which immunohistochemistry staining showed that intra-discal AOPPs loading caused up-regulated expression of p16 in AF tissues of degenerative discs. These findings indicated an endogenous pathogenic role of AOPPs in IVDD progression. We further confirmed that exposure of AF cells to AOPPs leads to increased senescence *in vitro*. Therefore, the increased AF senescence cells might be the pathology basis of AOPPs-induced IVDD.

NOX4, in contrast to all other isoforms of the NADPH oxidase family, is constitutively active and has been proved to be a major cause of NP cell senescence (Feng et al., 2017). Our previous study has shown that up-regulated expression of NOX4 was found in chondrocytes after AOPPs challenge *in vivo* and *in vitro* (Liao et al., 2020). However, there were no studies that elucidate the roles of NOX4 in the senescence of AF cells. In this study, both *in vivo* and *in vitro*, AOPPs were found to markedly up-regulate the expression of NOX4. Therefore, we firstly used Apocynin, a NADPH oxidase inhibitor, and Setanaxib, a potent dual NOX1/4 inhibitor, to pre-incubate AF cells before the AOPPs challenge. We found that both two inhibitors could reduce the pathological effects induced by AOPPs, indicating that the NOX family was involved in the pathological process. As NOX1 is mainly expressed in colon epithelium (Szanto et al., 2005), we hypothesized NOX4 was the major signal transduction molecule. Then we used two NOX4-specific shRNAs packaged by ADV, avoiding off-target effects, transfected AF primary cells. AOPPs-induced senescence was abrogated by NOX4-specific knockdown, indicating the cellular mechanisms were mediated specifically by NOX4. Finally, AAV that carried a shRNA-2 sequence that was previously validated was injected into rat caudal discs before stimulating the discs with RSA and AOPPs. Discs with blocked NOX4 expression had lower grades of degeneration and down-regulated expression of senescence markers and senescence-associated inflammatory proteins, suggesting an important role for NOX4 in disc degeneration caused by AOPPs.

The MAPK cascades constituting three kinase complexes, p38 MAPK, extracellular regulated kinase(ERK), and c-Jun N-terminal kinase(JNK), are substrates for phosphorylation by MAPK kinases (Kim & Choi, 2010). Our previous study reported the MAPK pathway was involved in AOPPs signal transduction (Liao et al., 2020; Zhu et al., 2018). In this experiment, all the three MAPK pathways were activated phosphorylation by AOPPs stimulation, and all the three MAPK inhibitors could block AOPPs-induced expression of p53, p21, p16, IL-1*β*, and TNF-*α* in AF cells. Hence, MAPK pathways were involved in AOPPs-triggered up-regulation of these senescence markers and senescence-associated inflammatory proteins. To identify the upstream signal of these MAPK, we tested MAPK phosphorylation expression of AF cells challenged by AOPPs that pre-incubated with APO, GKT, and two NOX4-specific shRNAs. AOPPs-triggered activation of MAPK pathways attenuated by both inhibitors (*in vitro*) and NOX4-specific knockdown (*in vitro* and *in vivo*), indicating upstream signal was mediated by NOX4.

IL-1*β* and TNF-*α* are highly expressed in both animal models and human aging and degenerative intervertebral discs (Le Maitre, Freemont & Hoyland, 2005; Bachmeier et al., 2007). They not only promote senescence of adjacent cells in degenerating discs but also increase oxidative stress within the disc (Feng et al., 2016). As a major pro-inflammatory factor, IL-1*β* recruited immune cells such as macrophages and neutrophils to the site of inflammation, releasing inflammatory mediators and exacerbating disc degeneration (Johnson et al., 2015). However, our results show that albumin, an essential nutrient, also accumulates in the degenerating disc, indicating that there is no shortage of raw materials for the production of AOPPs. Therefore, it can be predicted that when AOPPs start to appear in the degenerated disc tissue, it will activate the high expression of NOX4, induce AF cell senescence through the MAPK pathway, and continuously secrete IL-1*β* and TNF-*α*, which together with the activation of NOX4 aggravate oxidative stress on the one hand (Li et al., 2021), and recruit immune cells on the other, both of which work together to convert the accumulated albumin in the disc into AOPPs. A possible positive feedback loop is formed. In this positive feedback loop, we can easily find that the AOPPs/NOX4 pathway is the core part, and NOX4 is a key factor in the induction of downstream pathological effects by AOPPs. Our experimental results show that blocking the high expression of NOX4 could block most of the downstream pathological effects induced by AOPPs, thus this positive feedback loop can be blocked, oxidative stress can be controlled, and the rate of disc degeneration can be slowed down.

The main contribution of our study is the identification of the pathological role of the AOPPs/NOX4 pathway in AF cell senescence, and that blocking this pathway attenuate IVDD. Our limitation is that we did not use systemic knockout or conditional knockout mice for further validation, which is a direction we will continue to investigate in the future.

## Conclusions

AOPPs which are accumulated in the degenerative discs may increase the production of senescence markers and senescence-associated inflammatory proteins in AF cells via the NADPH oxidase 4-dependent, MAPK mediated pathway and eventually accelerate IVDD (Fig. 7G). Targeting at the pathophysiological effects of AOPPs related cellular mechanisms can attenuate AF cell senescence, delaying IVDD.

##  Supplemental Information

10.7717/peerj.13826/supp-1 Supplemental Information 1 ARRIVE 2.0 Checklist

## Additional Information and Declarations

### Data Availability

The following information was supplied regarding data availability: The raw data is available at Figshare: Dai, Xiangheng (2022): fig 1 Source data. figshare. Figure. https://doi.org/10.6084/m9.figshare.19596430.v3
Dai, Xiangheng (2022): fig 2 Source data. figshare. Figure. https://doi.org/10.6084/m9.figshare.19596439.v1
Dai, Xiangheng (2022): fig 3 Source data. figshare. Figure. https://doi.org/10.6084/m9.figshare.19596445.v1
Dai, Xiangheng (2022): fig 4 Source data. figshare. Figure. https://doi.org/10.6084/m9.figshare.19596448.v1
Dai, Xiangheng (2022): fig 5 Source data. figshare. Figure. https://doi.org/10.6084/m9.figshare.19596451.v1
Dai, Xiangheng (2022): fig 6 Source data. figshare. Figure. https://doi.org/10.6084/m9.figshare.19596457.v1
Dai, Xiangheng (2022): fig 7 Source data. figshare. Figure. https://doi.org/10.6084/m9.figshare.19596514.v1
